# Direct Synthesis of Partially Chain-Straightened Propylene Oligomers and P-MA Co-Oligomers Using Axially Flexible Shielded Iminopyridyl Palladium Complexes

**DOI:** 10.3390/polym15010111

**Published:** 2022-12-27

**Authors:** Huayin Sun, Huijun Fan, Chuangao Zhu, Wenping Zou, Shengyu Dai

**Affiliations:** 1 School of Chemical and Materials Engineering, Huainan Normal University, Huainan 232038, China; 2 Institutes of Physical Science and Information Technology, Key Laboratory of Structure and Functional Regulation of Hybrid Materials of Ministry of Education, Anhui University, Hefei 230601, China; 3 McKetta Department of Chemical Engineering, University of Texas at Austin, Austin, TX 78712, USA; 4 Jiangsu Key Laboratory of Advanced Catalytic Materials and Technology, Changzhou University, Changzhou 213164, China

**Keywords:** chain-straightened, propylene oligomers, co-oligomers, Pd(II) catalysts

## Abstract

In this study, a series of partially chain-straightened propylene oligomers and functional propylene–methyl acrylate (P-MA) co-oligomers were synthesized with 8-alkyl-iminopyridyl Pd(II) catalysts. The molecular weight and polar monomer incorporation ratio could be tuned by using Pd(II) catalysts with various 8-alkyl-naphthyl substituents (8-alkyl: H, Me, and n-Bu). In propylene oligomerization, all the 8-alkyl-iminopyridyl Pd(II) catalysts convert propylene to partially chain-straightened (119–136/1000 C) oligomers with low molecular weights (0.3–1.5 kg/mol). Among the catalysts, Pd1 with non-substituent (H) on the ligand showed the highest activity of 5.4 × 10^4^ g/((mol of Pd) h), generating oligomers with the lowest molecular weight (*M*_n_: 0.3 kg/mol). Moreover, polar-functionalized propylene-MA co-oligomers with very high incorporation ratios (22.8–36.5 mol %) could be obtained in the copolymerization using these 8-alkyl-iminopyridyl Pd(II) catalysts. Additionally, Pd1 exhibited the best performance in propylene-MA copolymerization as it displayed the highest MA incorporation ratio of up to 36.5 mol%. All the three catalysts are capable of generating partially chain-straightened P-MA co-oligomers and the activities decrease gradually while the molecular weight increases with the increasing steric hindrance of the alkyl substituent (H < Me < n-Bu). Compared to Pd4 with the rigid 8-aryl substituent, the flexible 8-alkyl-iminopyridyl Pd(II) catalysts (Pd1-3) not only showed much higher activities in the propylene oligomerization, but also yielded P-MA co-oligomers with significantly higher incorporation ratios in the propylene co-oligomerization.

## 1. Introduction

Since polypropylene was first synthesized in 1950s, this type of thermoplastic polymer has become one of the most used plastic products worldwide [1,2]. The excellent chemical and mechanical properties of polypropylene enable it to be used in packaging, manufacturing, the automotive industry, and household appliances [3]. Regardless of its widespread usage, the nonpolar properties of polypropylene limits its utilization. To improve the polarity of polypropylene for adhesion, dye-ability and compatibility, functional groups were always introduced into the material [4,5]. A post-modification technique was commonly used in industry. However, a deterioration of the accompanying properties always happened due to the processes occurring under harsh conditions, such as extreme heat, irradiation with high energy, and excessive etching. Additionally, the uncontrollable functional group distribution and side reactions would always diminish the material’s quality [4,5]. Therefore, propylene and functional vinyl monomers copolymerization using transition metal catalysts is the most straightforward and economic strategy to generate functional polypropylenes. Early-transition-metal catalysts exhibit highly efficient propylene polymerization. Additionally, Ziegler-Natta catalysts, metallocene catalysts, and other early-transition-metal catalysts were always easily deactivated by functional-group-containing polar monomers, although several cases have shown they could be applied in polar functional monomer and olefin copolymerization when the incorporated polar monomers were protected or tied up by Lewis acid as a “masking reagent” [6,7,8,9,10,11,12,13]. Recently, several cases showed a few cationic metallocene and (pyridylamido)Hf complexes could directly copolymerize propylene and polar monomers bearing the special structure without a “masking reagent” [14,15,16,17].

**Chart 1:**
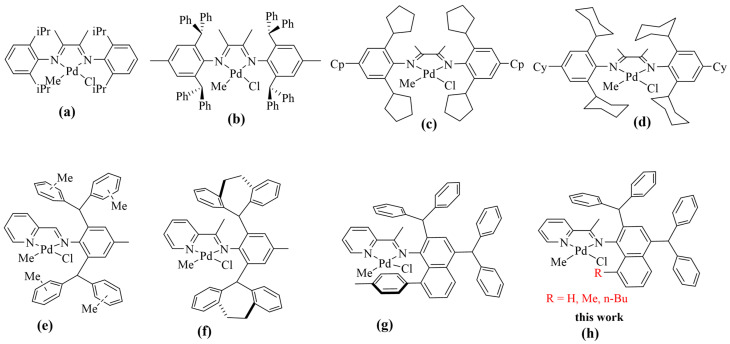
The α-diimine (**a**–**d**) and iminopyridyl (**e**–**h**) palladium complexes used for propylene (co)polymerization and this work (**h**).

In the last three decades, the development of late-transition-metal catalysts in olefin polymerization has been witnessed owing to their great capability of olefin–polar monomer copolymerization. Compared to early-transition metals, late-transition metal (palladium and nickel) complexes have higher polar functional group tolerance [18,19,20]. Since Brookhart et al. initially reported α-diimine bearing Ni(II) and Pd(II) complexes could be utilized in olefin (co)polymerization in the 1990s, a series of ligands with imine structures have been developed [21,22,23,24,25,26,27,28,29,30,31,32,33,34,35,36]. The polyolefin characters including molecular weight, polymer dispersity index (PDI), and microstructure not only depend on the polymerization conditions, but the coordinated complex ligands [37,38,39,40,41]. Among the α-diimine-based analogs, iminopyridyl-based Ni(II) and Pd(II) complexes have been noticed. The obtained oligomers using Ni(II) and Pd(II) catalysts bearing the iminopyridyl ligands have been reported in most ethylene (co)polymerizations [42,43,44,45,46,47,48]. When the ligands with an iminopyridyl structure are applied in propylene polymerization, the unilateral axial block provided by the imine motif would partially shield the metal ion center and result in the low molecular weight of the polymer [42,43,44,45,46,47,48]. Recently, we introduced a 1,5-di(dibenzosuberyl)amine structure into the iminopyridyl catalysts. The generated Ni(II)/Pd(II) complexes were apt to retard the chain transfer in polymerization. Therefore, the high-molecular-weight (co)polymers were produced [49,50,51,52]. We also found the Ni(II) and Pd(II) complexes bearing iminopyridyl ligands with dibenzhydryl and 8-alkyl/aryl-naphthyl substituents could induce ethylene (co)polymerization to form high-molecular-weight (co)polymers [52,53,54]. Moreover, these imine-based catalysts exhibited significant advantages in the copolymerization of propylene with polar comonomers and generated functionalized polypropylene materials (Chart 1) [55,56,57,58]. In this work, we describe the catalytic performance of propylene (co)polymerization using iminopyridyl Pd(II) catalysts (Chart 1h) composed of various 8-alkylnaphthyl substituents.

## 2. Experimental Section

### 2.1. General Procedures and Materials

All the reactions and polymerizations were carried out using standard Schlenk techniques or a glovebox under a dry nitrogen atmosphere. Anhydrous *n*-hexane, toluene, dichloromethane (DCM) and methyl acrylate were purchased from Ennegy Chemicals (Shanghai, China) and Sinopharm (Shanghai, China). All the solvents and monomers are dried with CaH_2_. The propylene gas was obtained from Nanjing Special Gas Co. (Nanjing, China) and applied in reaction without purification. The complexes Pd1-4 were synthesized according to the methods reported before [52,54]. The deuterated solvents were dried and distilled with CaH_2_. Nuclear magnetic resonance (NMR) spectra (^1^H: 600 MHz, ^13^C: 150 MHz) were recorded on a JNM-ECZ600R NMR instrument (Nippon Electronic Company, Tokyo, Japan) at ambient temperature unless otherwise stated. The chemical shifts observed in the ^1^H and ^13^C NMR spectra were referenced to the residual resonance of deuterated solvents (the coupling constants are reported in Hz, CDCl_3_: ^1^H, 7.26 ppm, ^13^C, 77.16 ppm). The molecular weight and molecular-weight distribution of polypropylene (PP), propylene oligomers, and propylene–MA co-oligomers (P–MA) were determined by size exclusion chromatography (SEC) analyses, which were carried out with two linear Styragel SEC columns containing Tosoh equipment (Tosoh Corporation, Tokyo, Japan) and eluted with THF. The system was calibrated with the polystyrene standards and operated at 40 °C at a flow rate of 0.35 mL/min with THF.

### 2.2. Procedures of Propylene (Co)Polymerization

#### 2.2.1. Propylene Polymerization 

A 350 mL thick-walled pressure vessel together with a magnetic stirrer was charged with 40 mL DCM in the glovebox. The pressure vessel was connected to a Schlenk line bearing high pressure. A solution of Pd1-4 (10 μmol) with NaBArF (20 μmol, 2.0 equiv.) in 2 mL of CH_2_Cl_2_ was introduced to the vessel via a syringe at room temperature. The reactor was pressurized and maintained at 4 atm propylene pressure with rapid stirring. The mixture was stirred for 3 h at the reaction temperature. After cooling down, the reactor was quenched in air. Then, the synthesized polymer was obtained via evaporation under vacuum until the weight remained constant. 

#### 2.2.2. Copolymerization of Propylene and MA

A 350 mL thick-walled pressure vessel with a stirring bar was charged with an appropriate amount of DCM, MA, and NaBArF in the glovebox. The pressure vessel was connected to a Schlenk line bearing high pressure. The Pd catalysts (20 μmol) in 2 mL CH_2_Cl_2_ were syringed into the well-stirred solution with the total reaction volume kept at 20 mL. The reactor was pressurized with propylene and maintained at 4 atm pressure. The copolymerization was terminated by passing through a pad of silica after continuous stirring for 12 h. Then, the formed copolymers were obtained under vacuum to an unchanged weight. X_MA_% = 3/(I_all_ − 3)×100%. I_all_: total H integral; I_OCH3_ = 3: OCH_3_ hydrogen integral (ca. 3.5–5.0 ppm). 

## 3. Results and Discussions

### Propylene Polymerization

**Chart 2:**
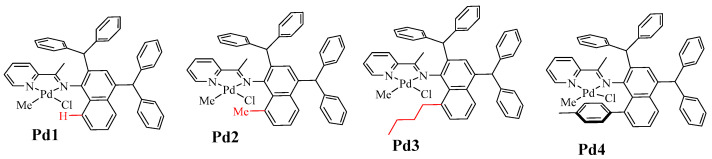
The iminopyridyl Pd(II) catalysts with various substituents at 8 position of naphthyl iminopyridyl ligands (Pd1: H; Pd2: Me; Pd3: butyl; Pd4: *p*-tolyl) were used for the propylene (co)polymerization.

**Figure 1:**
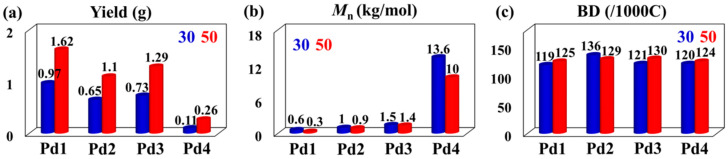
Comparisons on yield (**a**), molecular weight (**b**), and branching density (**c**) of propylene oligomers or polypropylene yielded with catalysts Pd1–Pd4 at 30 °C (blue) and 50 °C (red).

All of the Pd(II) complexes (Pd1-4) (Chart 2) were active in propylene polymerization after in situ dechlorination with 2.0 equiv. sodium tetrakis(3,5-bis(trifluoromethyl)phenyl)borate(NaBArF). The polymerization results are summarized in Table 1. All the 8-alkylnaphthyl Pd(II) complexes showed moderate catalytic activities (21.7–54 kg mol^−1^ h^−1^), and highly branched propylene oligomers (119–136/1000 C) with various molecular weights (0.3–1.5 kg mol^−1^) were obtained at given conditions (Table 1, Figure 1). Compared to Pd4 with the rigid 8-arylnaphthyl structure, Pd1-3 complexes bearing the 8-alkylnaphthyl structure with higher flexibility exhibited up to ten times higher activities with significantly lower molecular weights. This result is close to the ethylene polymerization results, in which palladium complexes bearing the 8-alkyl group also exhibited activities that were orders of magnitude higher [54]. The axial steric hindrance provided by the flexible substituents would probably facilitate the coordination and insertion of the propylene molecule [35,36,57]. The further comparison of Pd1, Pd2 and Pd3 revealed the higher molecular weight of propylene oligomers could be obtained using the complexes bearing the longer the alkyl group chain (entries 5–6 vs. 3–4 vs. 1–2, Table 1). This may be due to the axial steric effect. A more effective axial steric hindrance provided by longer alkyl substitutes would retard the chain transfer reaction more effectively in polymerization [38,54]. In addition, the activities of all the complexes, Pd14, increased 1.6–2.7 times when the polymerization temperature increased, while the molecular weight decreased (entries 1–8, Table 2). The significantly reduced energy barrier of propylene insertion into the active palladium species with these alkyl groups, and the increased ratio of chain transfer rate to chain growth rate are responsible for the results observed above.

**Figure 2:**
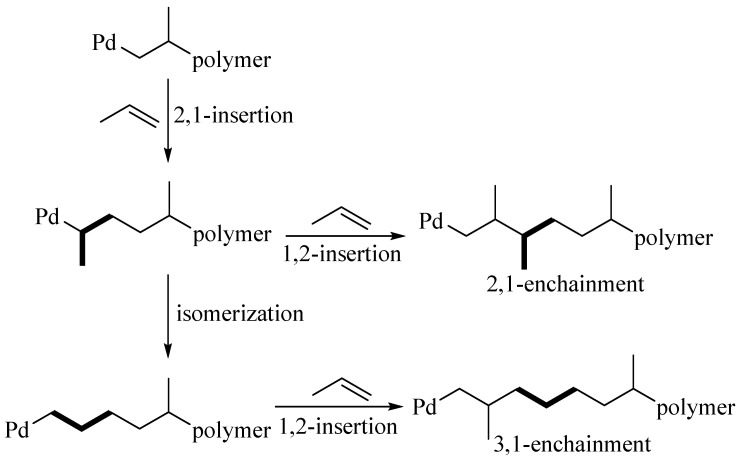
The general mechanism of propylene chain walking polymerization with iminopyridyl Pd(II) catalysts.

**Figure 3:**
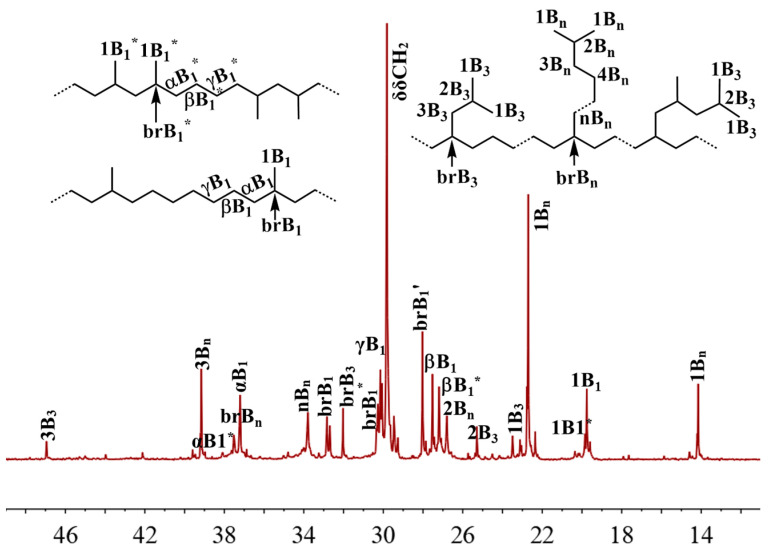
^13^C NMR spectrum analysis of the partially chain-straightened propylene oligomer obtained by using Pd3 at 30 °C (Table 1, entry 5). Branches are labeled as xBy, where y is the branch length and x is the carbon, starting from the methyl end with 1. The methine groups for the different branch lengths are labeled with brBy. The asterisk represents the situation when the methyl groups are in adjacent positions to each other.

Most interestingly, a chain walking phenomenon was also found in iminopyridyl palladium-catalyzed propylene polymerization (Figure 2). In contrast to the chain-walking-lead branch formation in ethylene polymerization, chain walking drives partial chain straightening in propylene polymerization (Figure 3). Theoretically, being derived from the methyl group containing monomer, polypropylene has a branching density of 333/1000 C. All the obtained propylene oligomers or polypropylenes in this study are 119–136/1000 C, which are significantly lower than 333/1000 C. This indicates that a remarkable chain straightening occurs during the polymerization. The calculated value of 1,3 chain straightening is around 0.49–0.54 (Table 1) for all the obtained propylene oligomers or polypropylenes, which indicates almost half of the propylene molecules experience 1,3 chain straightening. The chain walking in polymerization would facilitate the propylene insertion (Figures S1–S13). The driving force for chain walking is mainly due to the reduction in the steric hindrance during the insertion of propylene molecules in the polymerization process [59]. The ^13^C NMR spectrum analysis of a propylene oligomer yield with Pd3 at 30 °C is shown in Figure 3 [59,60]. Branch structures observed include methyl, 2-methylpropyl+, 2-methyl(CH_2_)n+, and 2,4-dimethylpentyl+. The adjacent-methyl and isolated-methyl branches are also observed.

The performance of the iminopyridyl Pd(II) complexes in propylene-MA copolymerization was also explored (Table 2). In this study, polar-functionalized propylene-MA co-oligomers with relatively high values of incorporation ratios (up to 36.5 mol %) were obtained (Table 2). The Pd(II) catalysts showed low activities (level of 10^3^ g mol^−1^ h^−1^), which were remarkably lower than those in propylene homo-polymerization. As expected, the higher MA concentration resulted in higher MA incorporation, but reduced activities. Compared to propylene homo-polymerization, the molecular weight was dramatically decreased when MA was supplied. However, the molecular weight was not further decreased with the extra MA supplement (Table 2, entries 1, 3, 5, 7 vs. 2, 4, 6, 8). The substantial reduction in molecular weights implied the insertion of excess polar monomers might greatly suppress the growth of the polymer chain and facilitate chain transfer during polymerization (Figures S14–S19) [49,52]. The propylene-MA co-oligomers obtained with Pd2-4 containing substituted alkyl/aryl group tended to produce co-oligomers with higher molecular weights compared to Pd1 (entries 1–2 vs. 3–8, Table 2). It demonstrates the steric effect is crucial for polymer chain propagation over chain transfer, which is similar to the propylene homo-polymerization result. The propylene-MA co-oligomer obtained with the 8-alkylnaphthyl Pd1-3 tended to generate co-oligomers with higher incorporation ratios compared to 8-arylnaphthyl Pd4 (entries 1–6 vs. 7–8, Table 2), which suggests the complexes with flexible alkyl substituents have the advantage of MA insertion.

## 4. Conclusions

In conclusion, we described propylene (co)polymerization using a series of 8-alkyl-1-naphthyl iminopyridyl Pd(II) catalysts. Compared to the exhibition of 8-arylnaphthyl Pd4 in propylene polymerization, all the 8-alkylnaphthyl Pd1-3 catalysts showed up to ten times higher activities, but generated propylene oligomers with significantly lower molecular weights. In the corresponding Pd(II)-catalyzed propylene-MA copolymerization, the 8-alkylnaphthyl Pd1-3 catalysts displayed higher MA incorporation ratios with similar molecular weights to 8-arylnaphthyl Pd4. Most notably, all the produced polypropylenes and propylene (co)oligomers are partial chain straightening, with almost half of the inserted propylene monomers undergoing 1,3 chain straightening. These propylene oligomers and propylene-MA co-oligomers could possibly be utilized as functional additives in surface modifiers or lubricants.

## Figures and Tables

**Table 1:** Effect of Catalysts and Temperatures on Propylene Polymerization. *^a^*.

Ent.	Precat.	*T*/°C	Yield/g	Act. *^b^*	*M*_n_ *^c^*	*M*_w_/*M*_n_*^c^*	*B ^d^*	[CH_3_]/[CH_2_] *^e^*	%1,3 *^f^*
1	Pd1	30	0.97	3.23	0.6	2.00	119	0.22	54
2	Pd1	50	1.62	5.40	0.3	1.27	125	0.23	53
3	Pd2	30	0.65	2.17	1.0	1.51	136	0.26	49
4	Pd2	50	1.10	3.67	0.9	1.53	129	0.24	51
5	Pd3	30	0.73	2.43	1.5	1.78	121	0.22	54
6	Pd3	50	1.29	4.30	1.4	1.59	130	0.24	51
7	Pd4	30	0.11	0.37	13.6	2.43	120	0.22	54
8	Pd4	50	0.26	0.87	10.0	1.57	124	0.23	53

*^a^* Reaction conditions: Pd catalyst (10 *μ*mol), NaBArF (2.0 equiv.), propylene (4 atm), CH_2_Cl_2_ (40 mL), polymerization time (3 h). *^b^* Activity = 10^4^ g/((mol of Pd) h). *^c^* *M*_n_ is in units of kg mol^−1^. Determined by SEC in THF at 40 °C vs. polystyrene standards. *^d^* B = Number of branches per 1000 C, as determined by ^1^H NMR spectroscopy. *^e^* Determined by ^1^H NMR spectroscopy. *^f^* 1,3-enchainment, calculated from the equation: %1,3-enchainment = [(1 − R)/(1 + 2R)] × 100, where R = [CH_3_]/[CH_2_].

**Table 2:** Propylene-MA copolymerization. *^a^*.

Ent.	Precat.	[MA]	Yield (g)	Act. *^b^*	*X*_MA_*^c^*(%)	*M*_n_ *^d^*	*M*_w_/*M*_n_*^d^*	*B ^e^*
1	Pd1	1	0.44	1.83	34.3	0.3	1.28	119
2	Pd1	2	0.37	1.54	36.5	0.3	1.28	152
3	Pd2	1	0.34	1.42	22.8	0.5	1.37	108
4	Pd2	2	0.22	0.92	25.1	0.5	1.21	154
5	Pd3	1	0.11	0.46	25.3	0.6	1.32	126
6	Pd3	2	0.09	0.38	33.8	0.5	1.20	142
7	Pd4	1	0.17	0.71	13.8	0.5	1.21	105
8	Pd4	2	0.13	0.54	23.0	0.5	1.16	151

*^a^* General conditions: Pd catalysts (20 μmol), NaBArF (2.0 equiv.), propylene (4 atm), polymerization time (12 h), the total volume of CH_2_Cl_2_ and MA (20 mL), polymerization temperature (40 °C), *^b^* Activity = 10^3^ g/((mol of Pd) h), *^c^* *X*_MA_ = Incorporation of MA, *^d^* *M*_n_ is in units of kg mol^–1^. Determined by SEC in THF at 40 °C vs. polystyrene standards, *^e^* B = Number of branches per 1000 C, as determined by ^1^H NMR spectroscopy. The branches ending with functional groups are added to the total branches.

## Data Availability

The data presented in this study are available on request from the corresponding author.

## Supplementary Materials

The following supporting information can be downloaded at: https://www.mdpi.com/article/10.3390/polym15010111/s1, Figures S1–S13: ^1^H and ^13^C NMR of some representative propylene oligomers and P-MA co-oligomers; Figures S14–S19: SEC of some representative propylene oligomers and P-MA co-oligomers.
