# Supplementary material for: Direct Synthesis of Partially Chain-Straightened Propylene Oligomers and P-MA Co-Oligomers Using Axially Flexible Shielded Iminopyridyl Palladium Complexes
Source: Polymers (Basel). 2022 Dec 27;15(1):111. doi: 10.3390/polym15010111 (PMC9823751; doi:10.3390/polym15010111)
Supplement: Supplementary file 1 [file polymers-15-00111-s001.zip › polymers-2087093-supplementary.pdf]

Direct Synthesis of Partially Chain-Straightened Propylene Oligomers and P-MA Co-Oligomers Using Axially Flexible Shielded Iminopyridyl Palladium Complexes

Huayin Sun [1], Huijun Fan [2], Chuangao Zhu [1], Wenping Zou [3,*] and Shengyu Dai [2,4,*]

1 School of Chemical and Materials Engineering, Huainan Normal University, Huainan 232038, China
2 Institutes of Physical Science and Information Technology, Key Laboratory of Structure and Functional Regulation of Hybrid Materials of Ministry of Education, Anhui University, Hefei 230601, China
3 McKetta Department of Chemical Engineering, University of Texas at Austin, Austin, TX 78712, USA
4 Jiangsu Key Laboratory of Advanced Catalytic Materials and Technology, Changzhou University, Changzhou 213164, China
* Correspondence: wenping@utexas.edu (W.Z.); daiyu@ustc.edu.cn (S.D.)

1. Spectra Data

1.1 ^{1}H and ^{13}C NMR of Some Representative Propylene Oligomers and P-MA Co-oligomers.

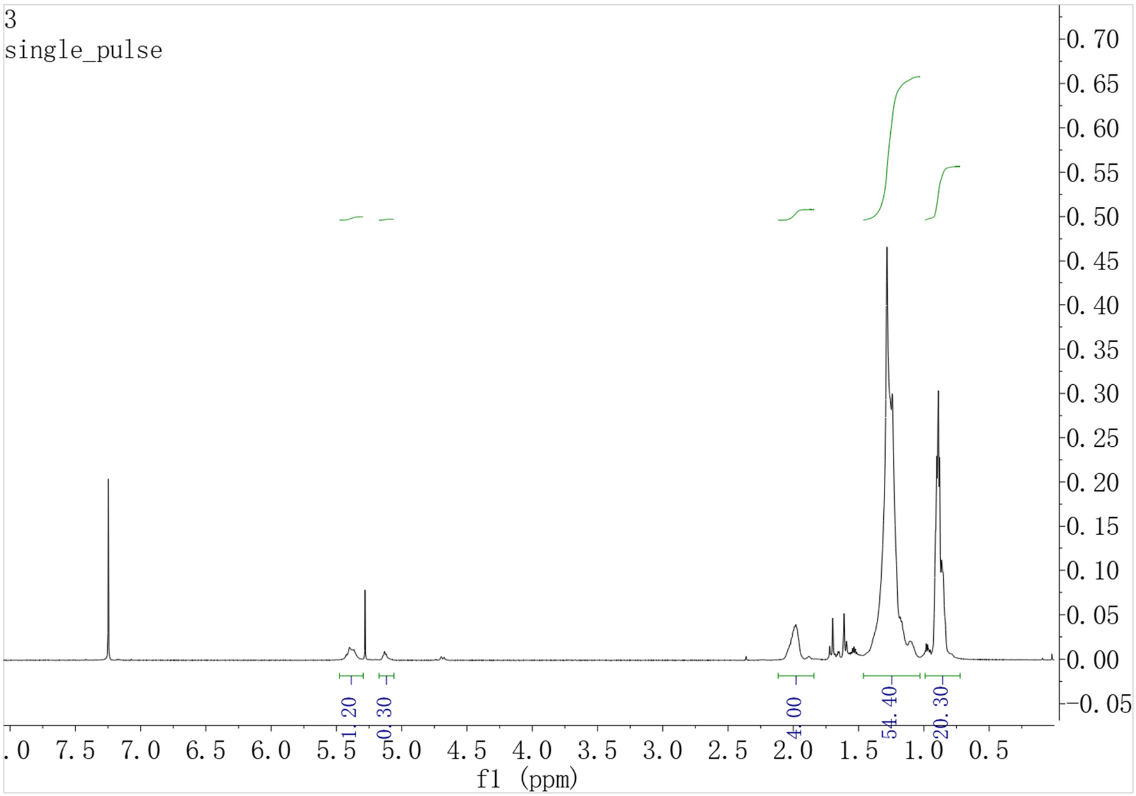

Figure S1. ^{1}H NMR spectrum of the propylene oligomer from table 1, entry 1.

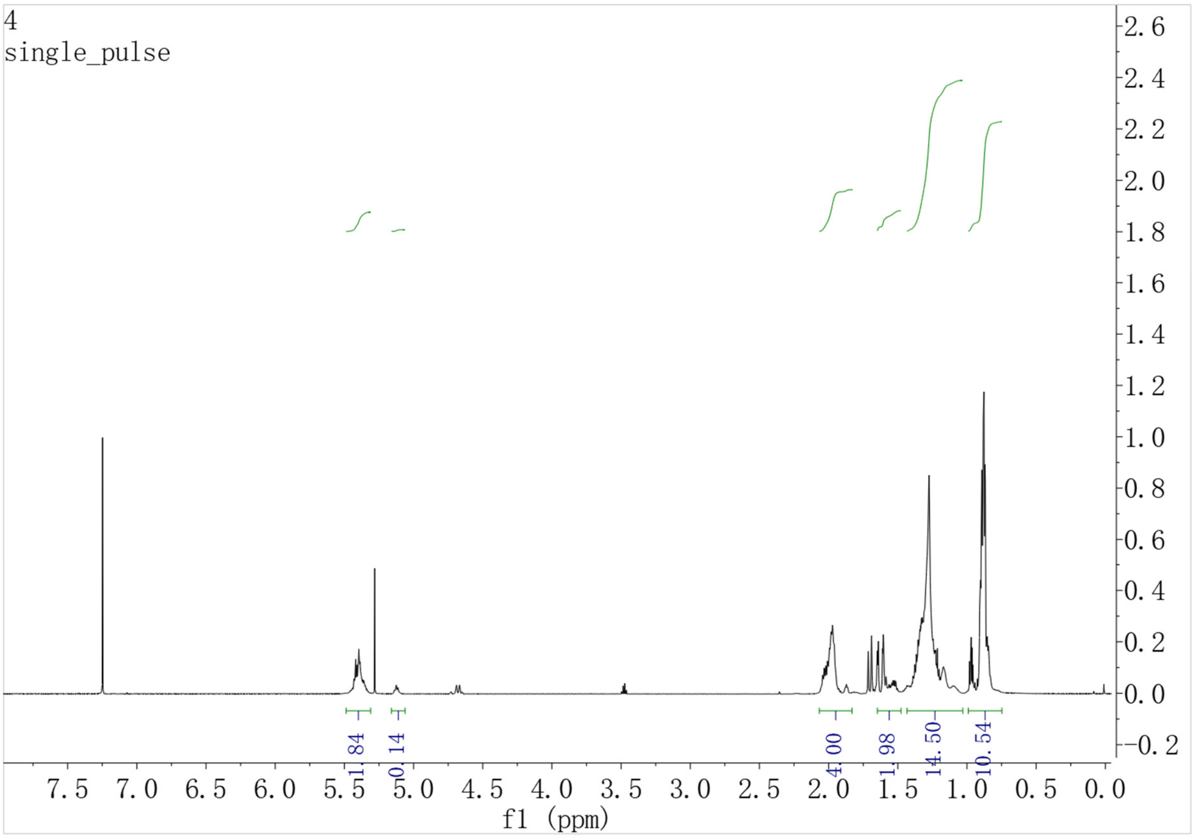

Figure S2. ^{1}H NMR spectrum of the propylene oligomer from table 1, entry 2.

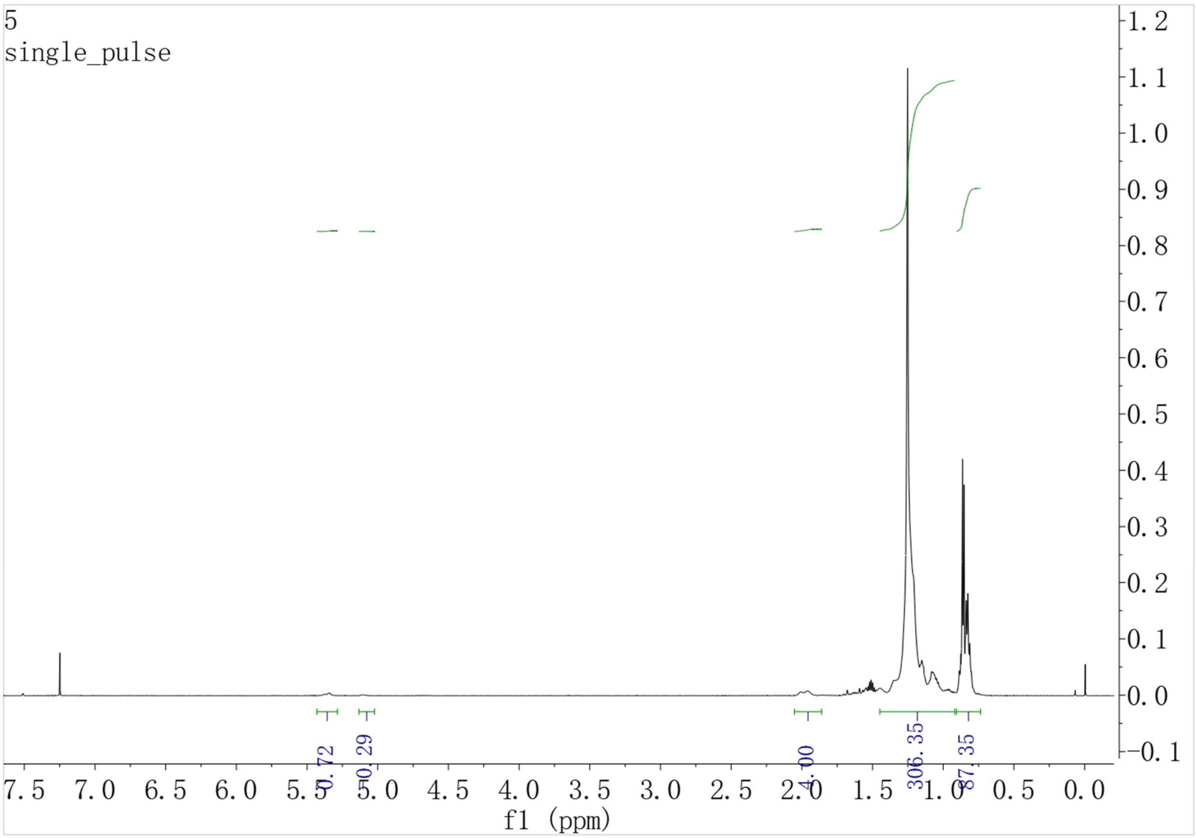

Figure S3. ^{1}H NMR spectrum of the propylene oligomer from table 1, entry 3.

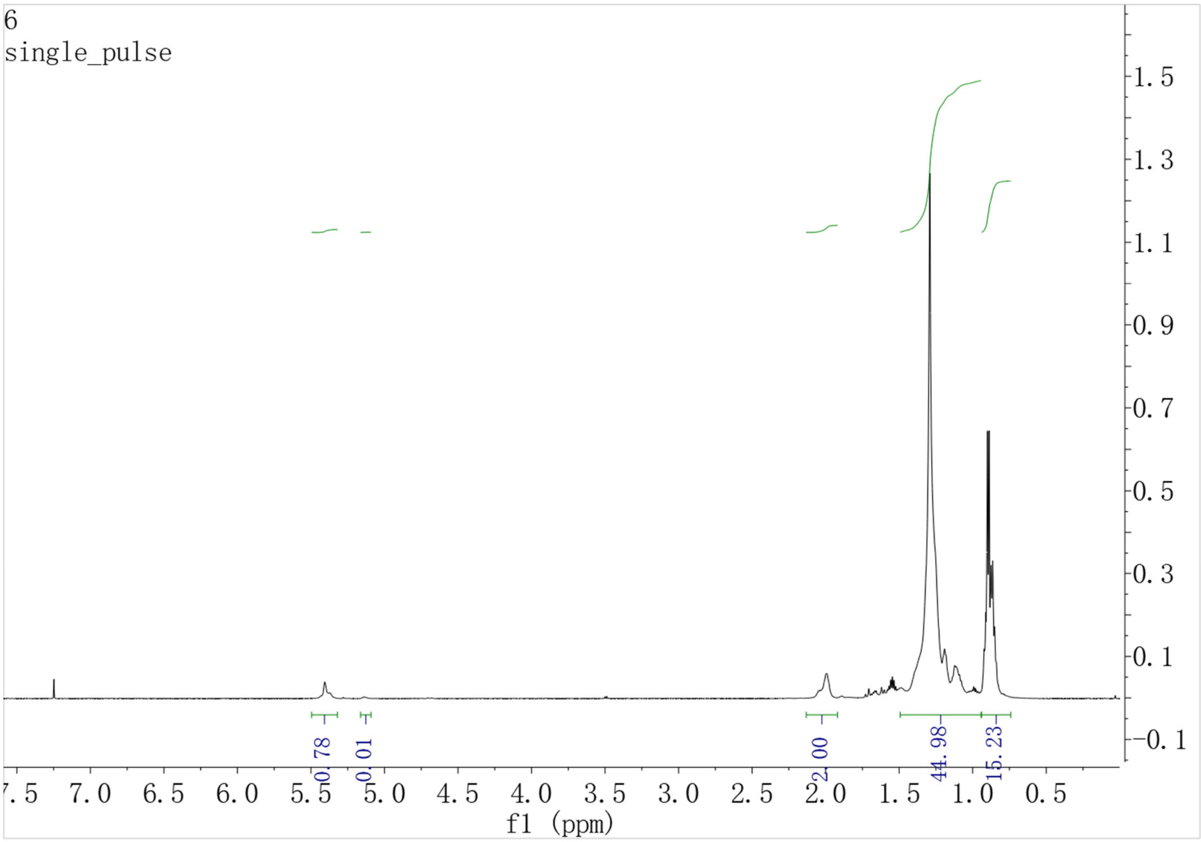

Figure S4. ^{1}H NMR spectrum of the propylene oligomer from table 1, entry 4.

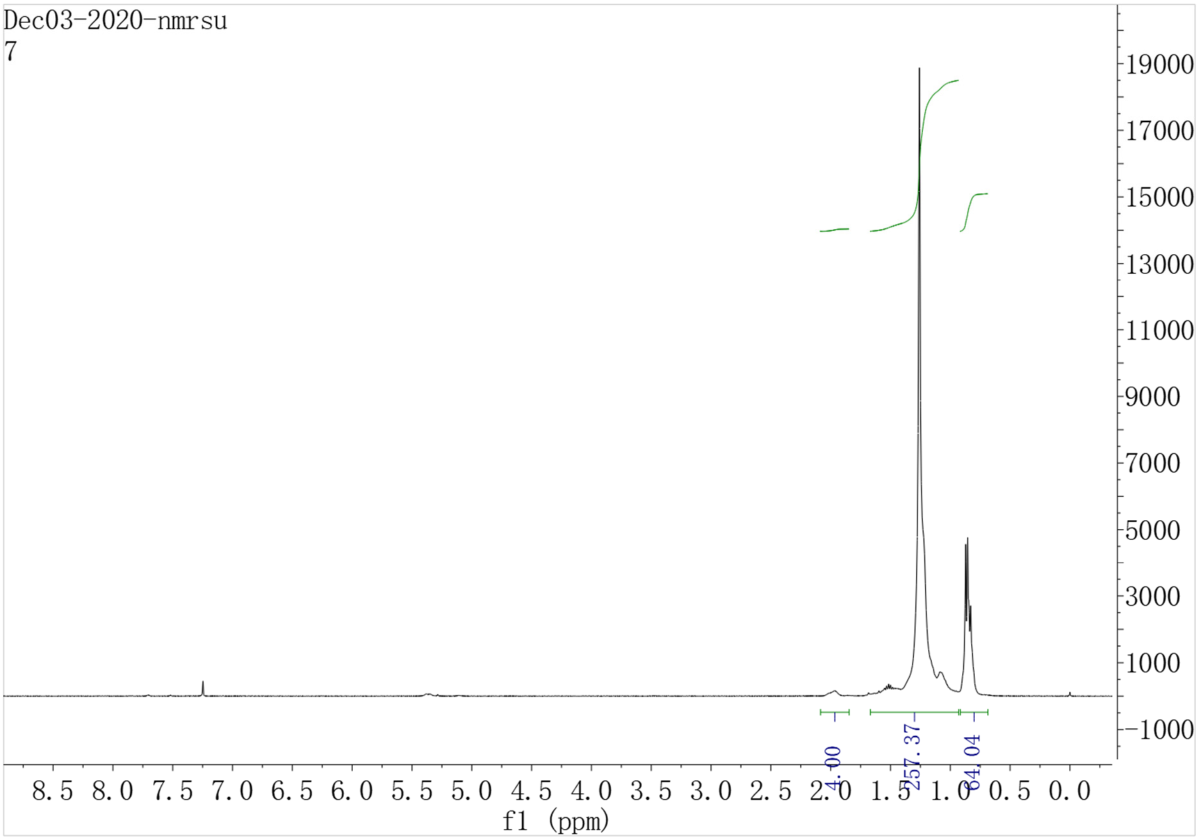

Figure S5. ^{1}H NMR spectrum of the propylene oligomer from table 1, entry 5.

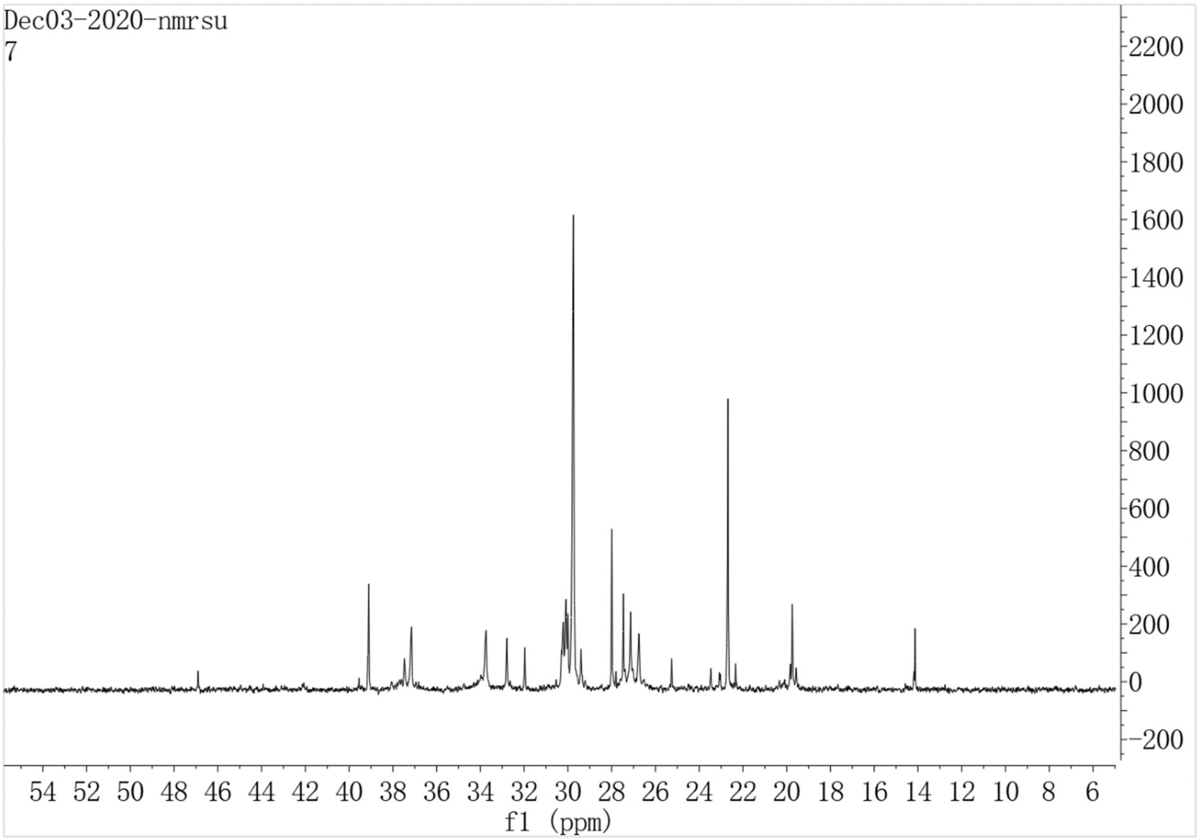

Figure S6. ^{13}C NMR spectrum of the propylene oligomer from table 1, entry 5.

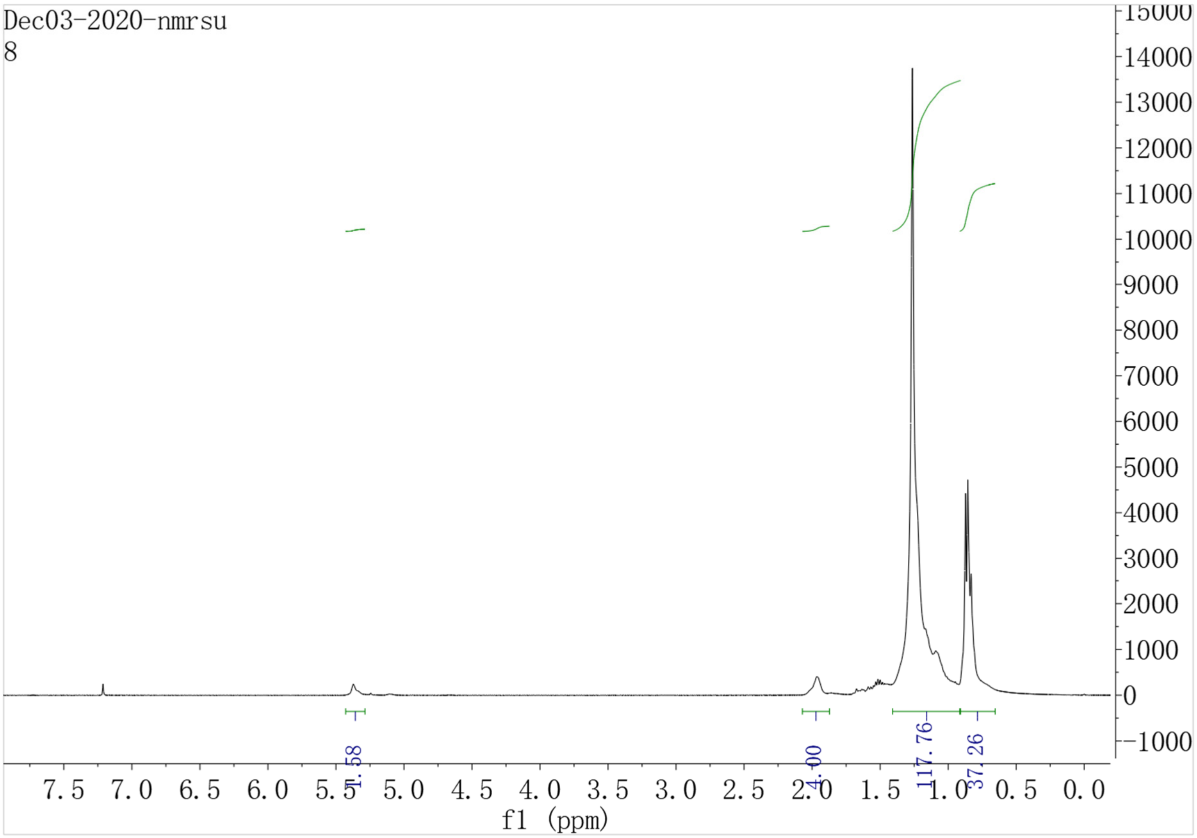

Figure S7. ^{1}H NMR spectrum of the propylene oligomer from table 1, entry 6.

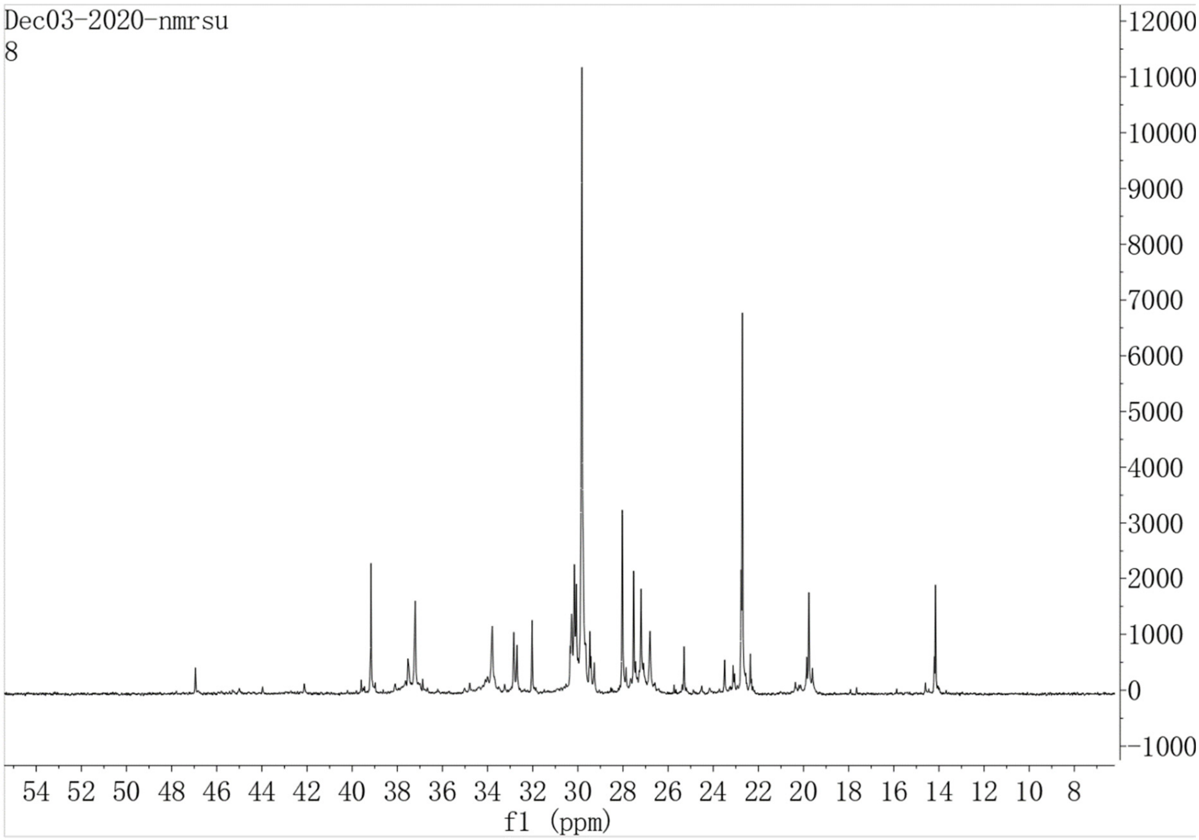

Figure S8. ^{13}C NMR spectrum of the propylene oligomer from table 1, entry 6.

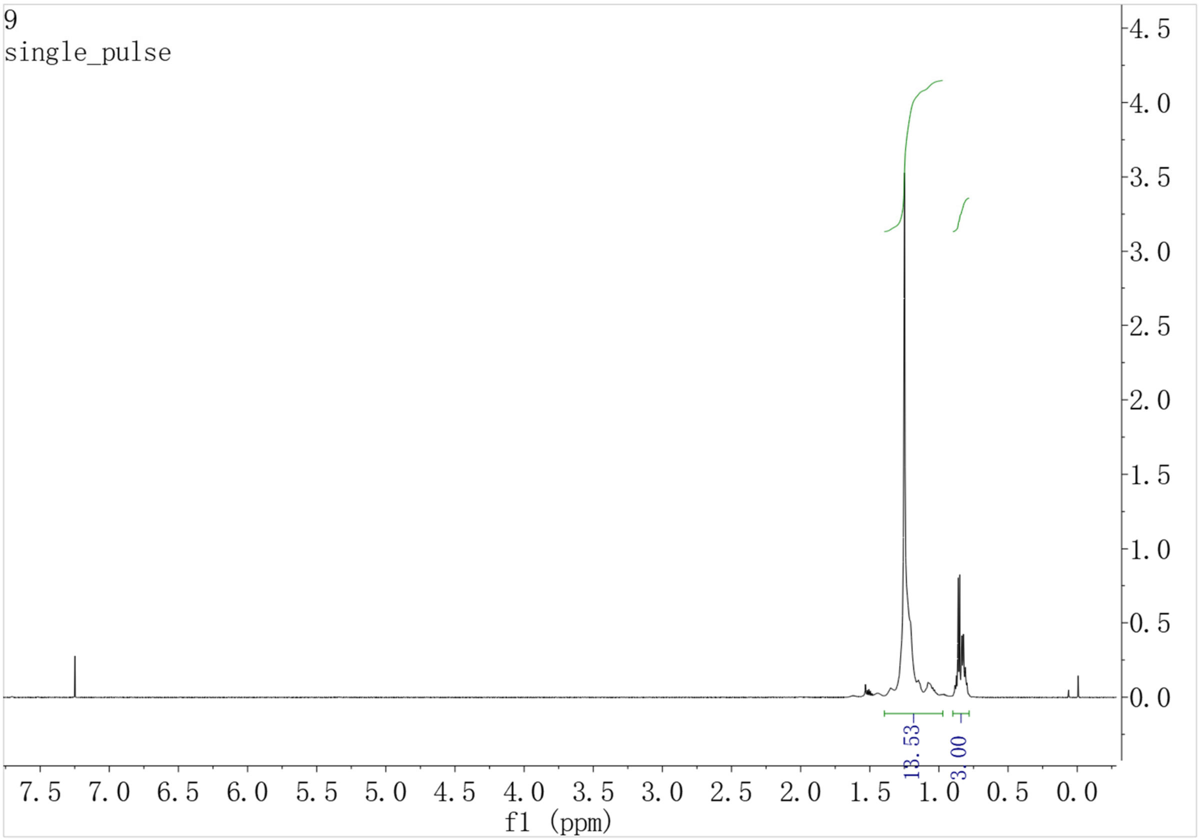

Figure S9. ^{1}H NMR spectrum of the polypropylene from table 1, entry 7.

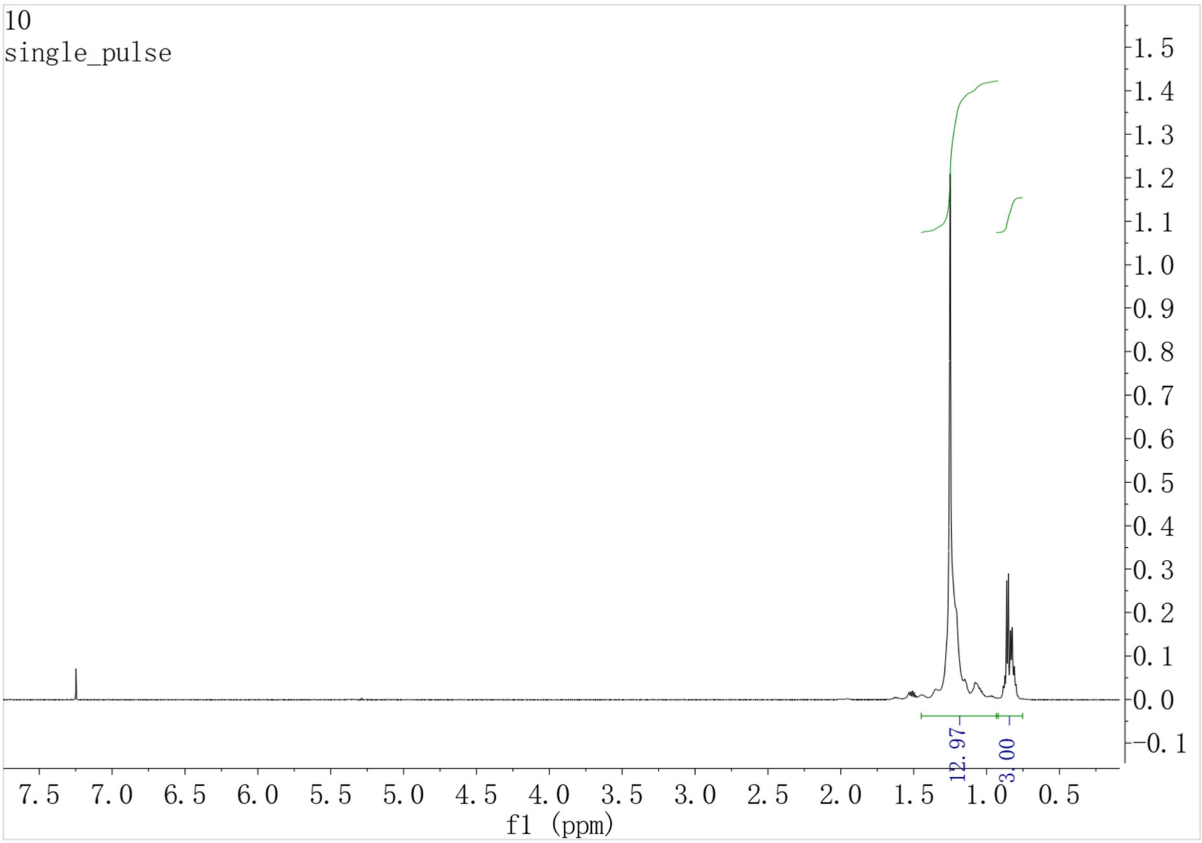

Figure S10. ^{1}H NMR spectrum of the polypropylene from table 1, entry 8.

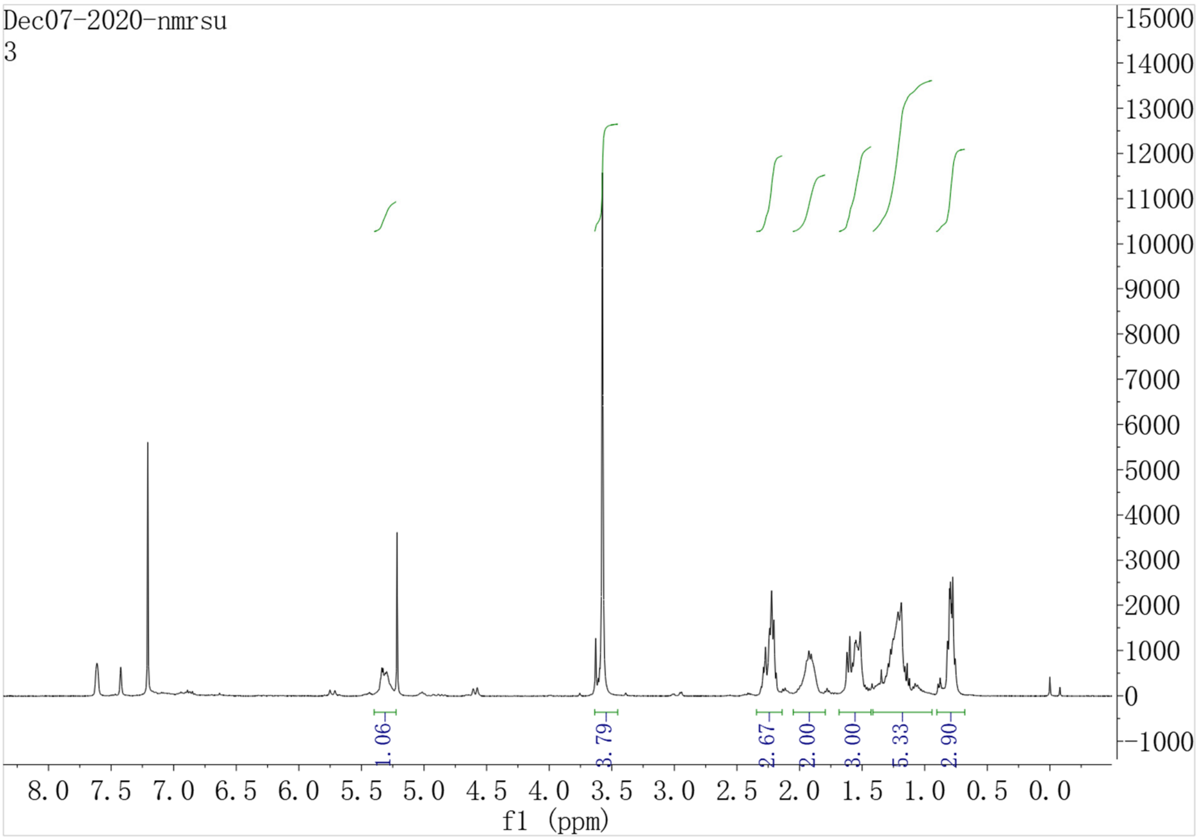

Figure S11. ^{1}H NMR spectrum of the P-MA co-oligomer from table 2, entry 1.

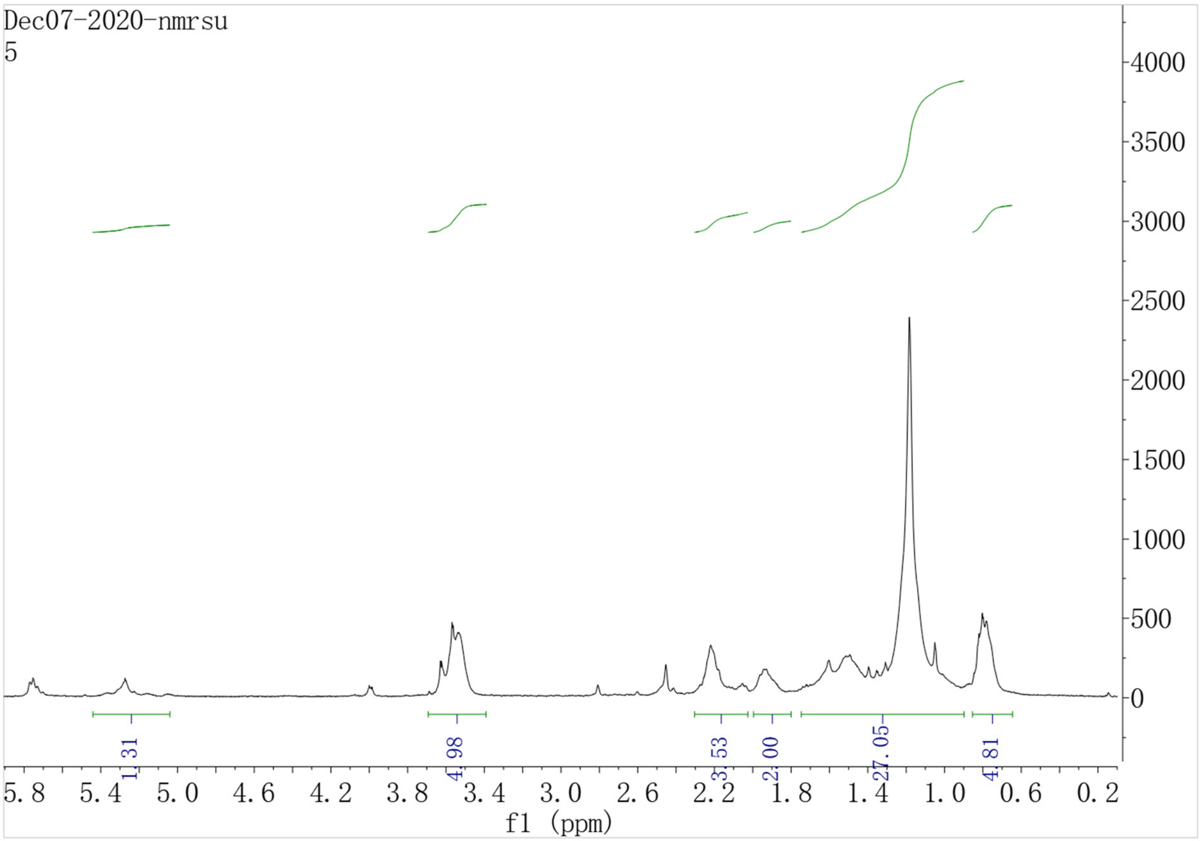

Figure S12. ^{1}H NMR spectrum of the P-MA co-oligomer from table 2, entry 3.

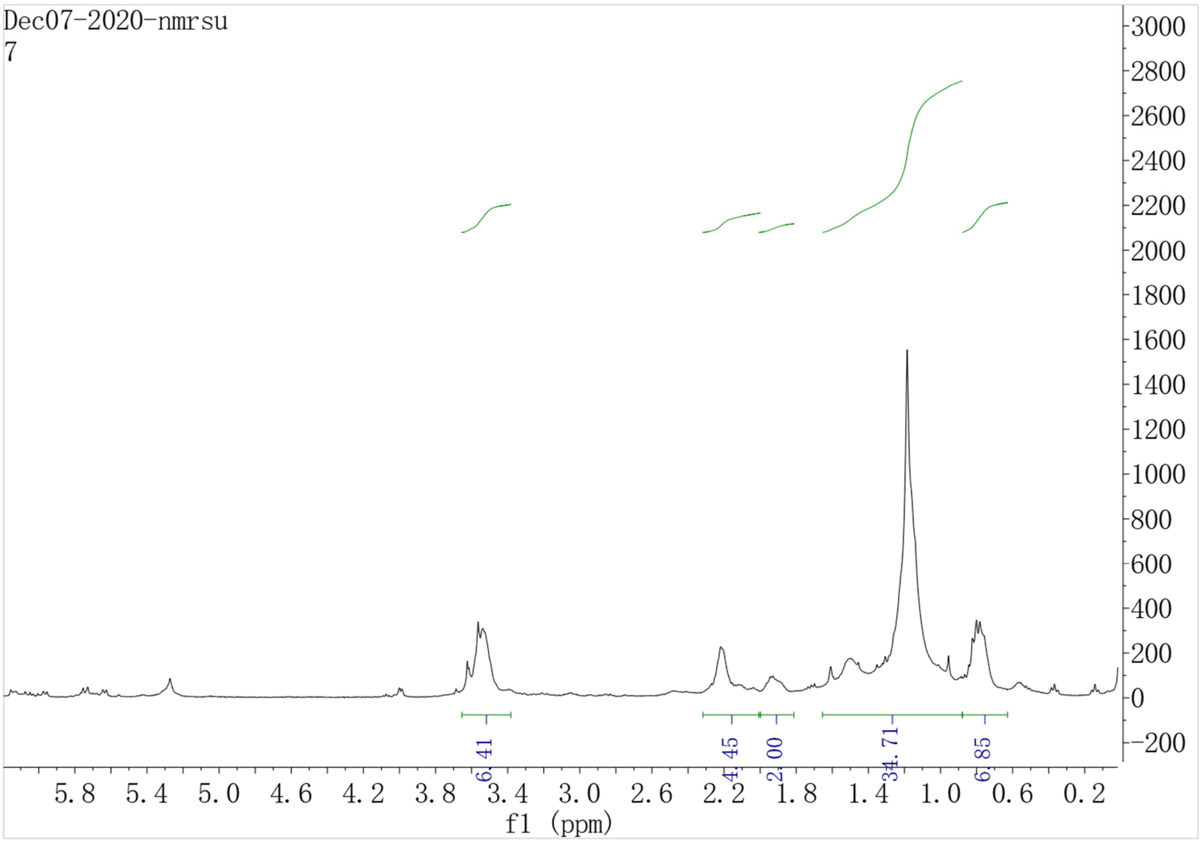

Figure S13. ^{1}H NMR spectrum of the P-MA co-oligomer from table 2, entry 5.

1.2 SEC of Some Representative Propylene Oligomers and P-MA Co-oligomers.

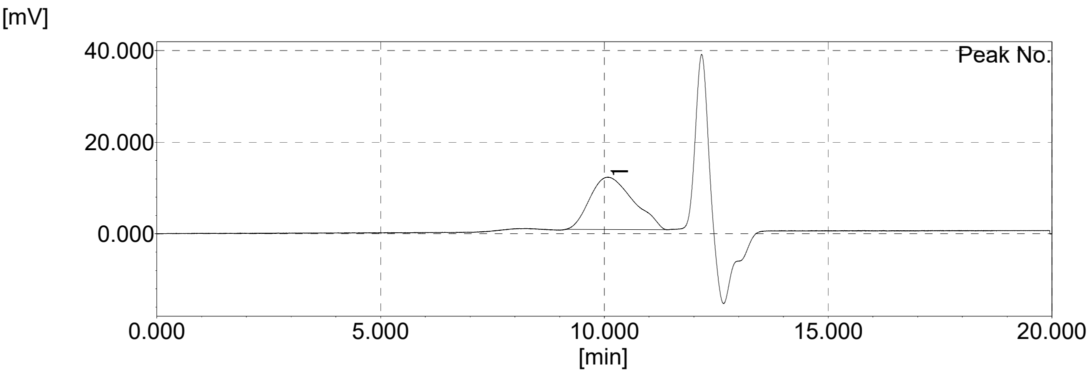

Result of molecular weight calculation (RI)

Peak 1 Base Peak

	[min]	[mV]	[mol]		
				Mn	1,036
Peak start	9.118	0.956	6,640	Mw	1,563
Peak top	10.072	12.395	1,590	Mz	2,161
Peak end	11.397	0.898	218	Mz+1	2,730
				Mv	1,563
Height [mV]			11.463	Mp	1,591
Area [mV*sec]			772.999	Mz/Mw	1.383
Area% [%]			100.000	Mw/Mn	1.508
[eta]			1562.59726	Mz+1/Mw	1.747

Figure S14. SEC of the propylene oligomer from table 1, entry 3.

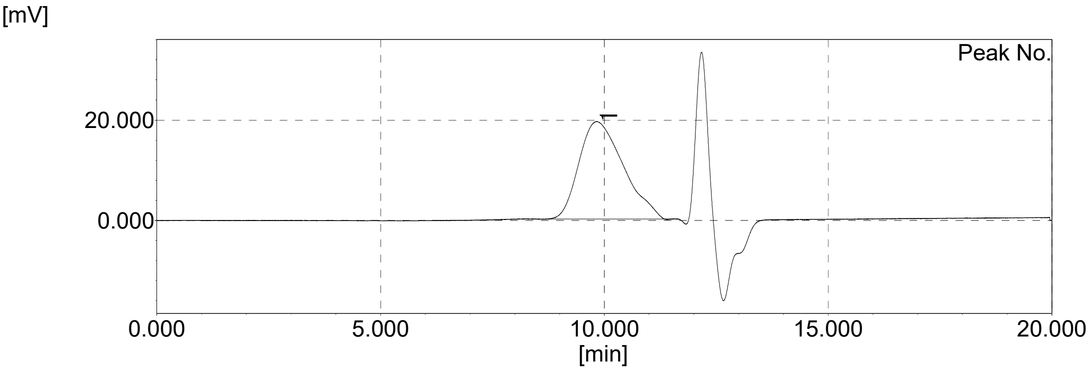

Result of molecular weight calculation (RI)

Peak 1 Base Peak

	[min]	[mV]	[mol]		
				Mn	1,397
Peak start	8.745	0.279	11,620	Mw	2,220
Peak top	9.832	19.722	2,279	Mz	3,143
Peak end	11.517	0.307	182	Mz+1	4,052
				Mv	2,220
Height [mV]			19.432	Mp	2,279
Area [mV*sec]			1336.045	Mz/Mw	1.416
Area% [%]			100.000	Mw/Mn	1.589
[eta]			2219.67740	Mz+1/Mw	1.825

Figure S15. SEC of the propylene oligomer from table 1, entry 6.

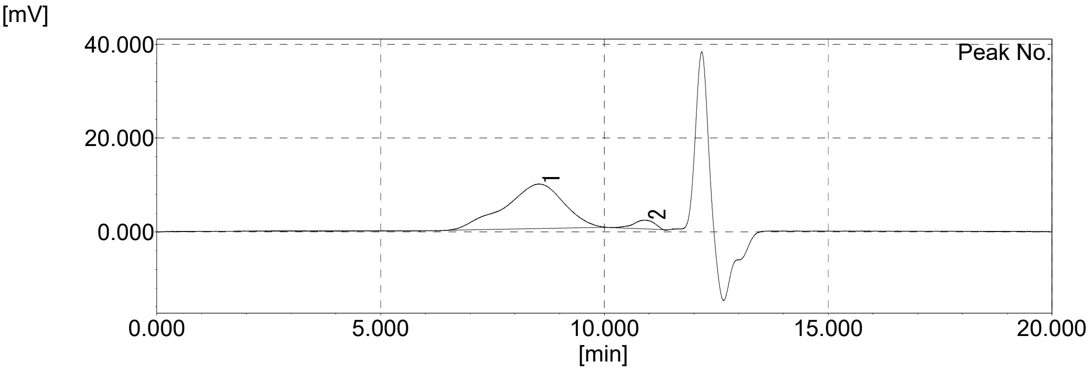

Result of molecular weight calculation (RI)

Peak 1 Base Peak

	[min]	[mV]	[mol]		
				Mn	13,572
Peak start	6.462	0.313	356,251	Mw	32,921
Peak top	8.543	10.234	15,722	Mz	77,329
Peak end	10.077	0.961	1,578	Mz+1	128,529
				Mv	32,921
Height [mV]			9.548	Mp	15,723
Area [mV*sec]			876.898	Mz/Mw	2.349
Area% [%]			93.393	Mw/Mn	2.426
[eta]			32920.53959	Mz+1/Mw	3.904

Figure S16. SEC of the polypropylene from table 1, entry 7.

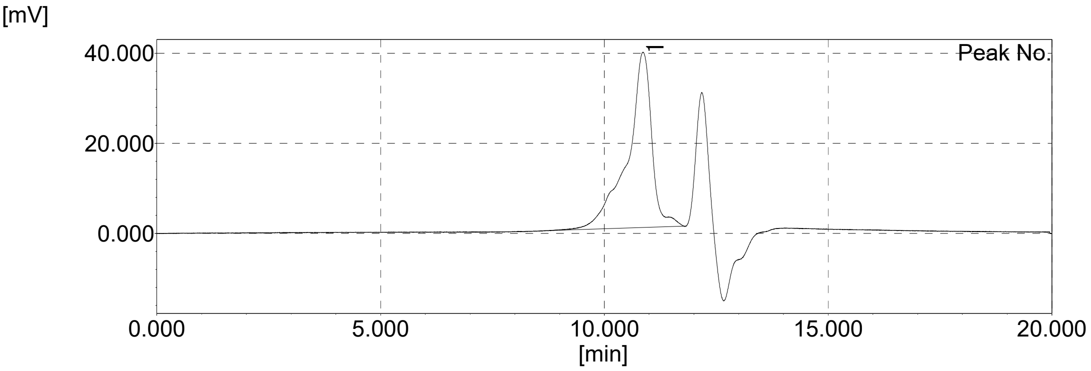

Result of molecular weight calculation (RI)

Peak 1 Base Peak

	[min]	[mV]	[mol]		
				Mn	540
Peak start	8.787	0.670	10,917	Mw	741
Peak top	10.865	40.257	484	Mz	1,194
Peak end	11.830	1.635	113	Mz+1	2,190
				Mv	741
Height [mV]			38.928	Mp	484
Area [mV*sec]			1467.768	Mz/Mw	1.610
Area% [%]			100.000	Mw/Mn	1.373
[eta]			741.29876	Mz+1/Mw	2.955

Figure S17. SEC of the P-MA co-oligomer from table 2, entry 3.

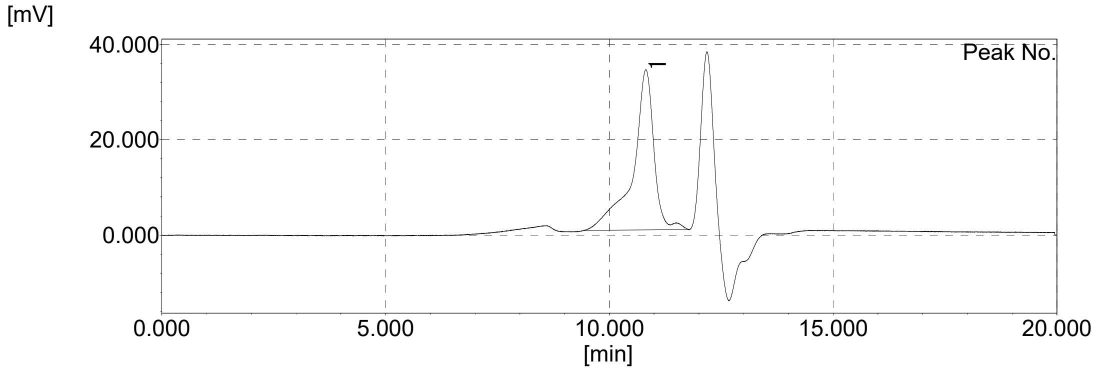

Result of molecular weight calculation (RI)

Peak 1 Base Peak

	[min]	[mV]	[mol]
Peak start	9.448	0.997	4,049
Peak top	10.808	34.676	527
Peak end	11.787	1.198	121
Height [mV]			33.562
Area [mV*sec]			1099.336
Area% [%]			100.000
[eta]			739.09892

Mn	562
Mw	739
Mz	1,063
Mz+1	1,538
Mv	739
Mp	527
Mz/Mw	1.439
Mw/Mn	1.316
Mz+1/Mw	2.080

Figure S18. SEC of the P-MA co-oligomer from table 2, entry 5.

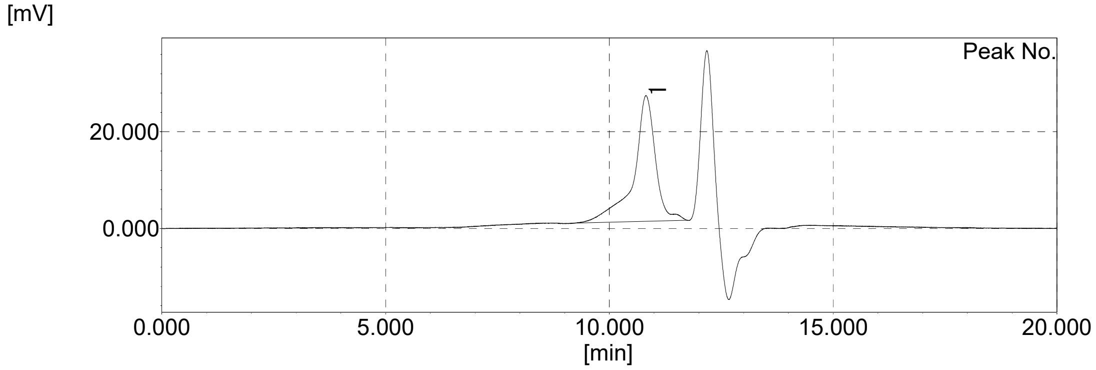

Result of molecular weight calculation (RI)

Peak 1 Base Peak

	[min]	[mV]	[mol]
Peak start	9.822	1.472	2,313
Peak top	10.890	19.865	466
Peak end	11.743	2.201	129
Height [mV]			17.988
Area [mV*sec]			617.993
Area% [%]			100.000
[eta]			576.07861

Mn	476
Mw	576
Mz	709
Mz+1	881
Mv	576
Mp	466
Mz/Mw	1.230
Mw/Mn	1.210
Mz+1/Mw	1.530

Figure S19. SEC of the P-MA co-oligomer from table 2, entry 7.
